# Papillary renal cell carcinoma in two young adults with glycogen storage disease type Ia

**DOI:** 10.1002/jmd2.12096

**Published:** 2020-01-29

**Authors:** Ariane Perry, Claire Douillard, Frederic Jonca, Francois Glowacki, Xavier Leroy, Paul Caveriviere, Aurélie Hubert, Philippe Labrune

**Affiliations:** ^1^ APHP, Hôpitaux Universitaires Paris Sud, Hôpital Antoine Béclère, Centre de référence des maladies héréditaires du métabolisme hépatique Clamart France; ^2^ Lille University Hospital, Hôpital Jeanne de Flandres, Centre de référence des maladies héréditaires du métabolisme Lille France; ^3^ Clinique Ambroise Paré Toulouse France; ^4^ Nephrology Department Huriez Hospital, Lille University Hospital Lille France; ^5^ Department of Pathology Univ. Lille, CHU Lille France; ^6^ Anatomy and pathology laboratory, les Feuillants Toulouse France; ^7^ Université Paris Sud Paris Saclay and INSERM U 1195 France

**Keywords:** glycogen storage disease, kidney, papillary renal carcinoma

## Abstract

Glycogen storage disease type Ia (GSD Ia) is a rare metabolic disease due to glucose‐6‐phosphatase deficiency. Chronic kidney disease is a frequent complication that may manifest itself by glomerular lesions and tubular dysfunction from the second decade of life. We report two young GSDIa patients with malignant renal tumor. The first patient was a 25‐year‐old man. He had chronic metabolic imbalance without kidney involvement. The tumor, a type 2 papillary renal carcinoma, was accidentally discovered during follow‐up. The second patient was a 27‐year‐old woman with chronic metabolic imbalance and chronic kidney involvement. The tumor, a grade 2 papillary carcinoma, was accidentally discovered during follow‐up. These two observations are, to date, the first to be reported. We suggest that annual monitoring of kidney imaging in GSDI patients should be systematic to detect renal cancer, from the second decade of life.

## 1 INTRODUCTION

Glycogen storage disease type I (GSDI), OMIM 232200 (Ia), and 232220 (Ib), is a rare inborn disease of hepatic and renal glycogen metabolism. The annual incidence of the disease is around 1:100 000 births. It is transmitted as an autosomal recessive disease, and is due to mutations in the G6PC gene (GSDIa, 80% of patients), or in the SLC37A4 gene (GSDIb) for the remaining 20%. It results from a defect in the glucose‐6‐phosphatase (G6Pase) system.

When the disease is not well‐controlled, chronic acidosis due to hyperlactacidemia, hyperuricemia, and hypertriglyceridemia can be severe, and are likely to facilitate the development of other manifestations, such as liver or renal complications. Development of liver adenomas is common with increasing age, and several cases of malignant transformations have been previously described.

Renal manifestations begin early in childhood with hyperperfusion and hyperfiltration, but remain asymptomatic for many years. Their mechanisms are multifactorial, due to glycogen deposition in kidney, and to metabolic imbalance. Worsening of renal function occurs in the second decade of life, and then can progressively evolve to renal failure. Glomerular lesions with proteinuria, proximal tubular dysfunction (with hyperphosphaturia, aminoaciduria, and glycosuria), and distal tubular dysfunction (with hypocitraturia and hypercalciuria), can occur. Renal cysts have been recently described, in both mice and humans.^1^, ^2^, ^3^, ^4^ Renal biopsies, when performed, revealed tubular atrophy, glomerulosclerosis, and interstitial fibrosis. Recent molecular studies suggest the involvement of the renin‐angiotensin system in the development of renal fibrosis and renal oxidative stress in total *G6pc* knockout mice.^5^

Here, we report two cases of renal tumor in two young GSD Ia patients.

## 2 PATIENT 1

The first patient is a 25‐year‐old Caucasian man. Glycogen storage disease type Ia was diagnosed during infancy (fasting hypoglycemia and hepatomegaly), and confirmed by molecular diagnosis (compound heterozygosity for both c.527A>G and c.1039 C>T mutations in the glucose 6 phosphatase gene).

This patient also has both Willebrand and Gilbert diseases. He had been treated with growth hormone at 12 years of age, for 1 year, for retarded growth, with no clinical improvement. He had been an active smoker since the age of 15 years, and recently quit smoking.

He used to have chronic metabolic imbalance during the second decade of life. Supplementations with uncooked cornstarch since infancy, then enteral nutrition by the age of 17 were not well tolerated because of chronic diarrhea. Blood tests performed at 22 were disturbed as follow: triglyceridemia 19 mmoL/L, uricemia 0.498 mmoL/L, bicarbonate 16 mmoL/L, lactic acid before breakfast 9 mmoL/L. The patient slowly developed hepatic polyadenomatosis. He also developed osteoporosis.

When he was 22 years old, uncooked cornstarch (300‐420 g/day) was replaced by Glycosade and oral fenofibrate was started. His diet was hyperglucidic, glucides representing 63% of caloric intake. This treatment allowed an improvement of metabolic balance. His metabolic blood tests between 22 and 25 years of age were as follow: triglyceridemia between 2.28 and 4.56 mmoL/L, uricemia between 0.4 and 0.5 mmoL/L, lactic acid before breakfast between 3 and 5 mmoL/L, bicarbonate between 20 and 24 mmoL/L. Hepatic polyadenomatosis remained stable on regular MRI follow‐up since the age of 22.

He had no renal disease. Proteinuria and microalbuminuria were negative, creatinine clearance was 120 mL/min/1.73 m^2^ (Cockroft‐Gault), calciuria was low. It is noteworthy that renal cysts had never been observed, either on MRI or on ultrasound examination.

He was referred when he was 25 year‐old in our center for his annual follow‐up. His metabolic balance was good and his blood tests were as follow: creatinine clearance 123 mL/min (Cockroft‐Gault), proteinuria <0.02 g/L, microalbuminuria 13.6 mg/L, uric acid 0.438 mmoL/L, triglyceridemia 2.52 mmoL/L, cholesterol 5.07 mmoL/L, hemoglobin 141 g/L, C reactive protein <5 mg/L, aspartate amino transferase 0.54 μKat/l (32 UI/l), alanine amino transferase 0.38 μKat/l (23 UI/l), GGT 0.69 μKat/l (41 UI/l). Glycemias were normal during 24 hours before and after meals, lactic acid were measured between 1.7 and 3.1 mmoL/L during 24 hours.

Hepatic MRI showed multiple adenomas and decrease in size of the largest adenoma (41 mm vs 48 mm the year before).

**Figure 1:**
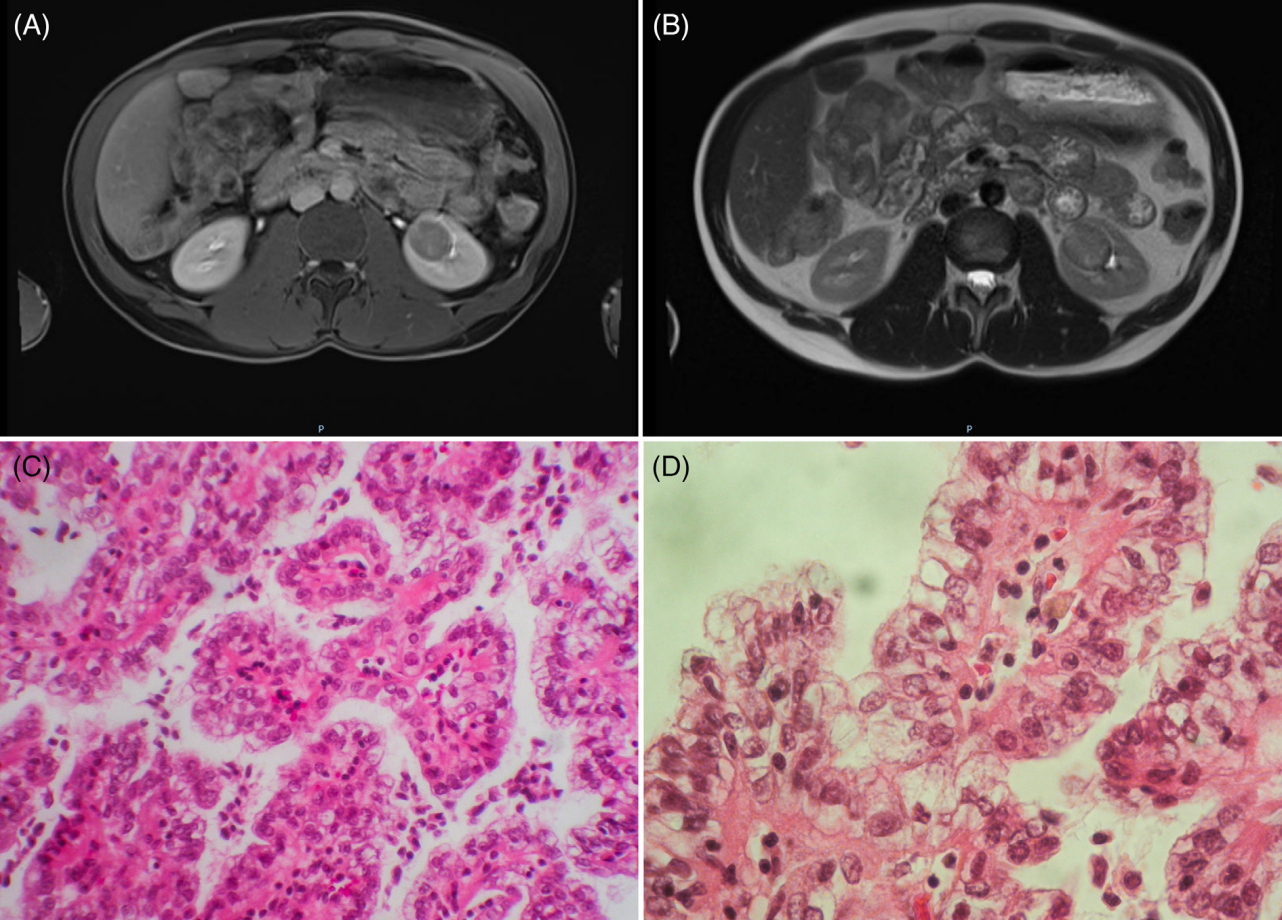
A, Renal MRI, T1 weighted image, with injection. Nodular lesion, isointense. B, Renal MRI, T2 weighted image, with injection. Nodular lesion, discreetly hyperintense with discreet contrast enhancement after injection. C, Papillary renal cell carcinoma type 2, HE. D, Papillary renal cell carcinoma type 2, HE

Renal MRI showed the appearance of a nodular lesion, at the lower pole of left kidney. The lesion diameter was 28 mm. On T1 weighted images, the lesion was isointense. On T2 weighted images, it was discretely hyperintense, with discrete contrast enhancement after injection (Figure 1A,B).

The patient underwent partial nephrectomy. The diagnosis of papillary renal cell carcinoma type 2 (grad ISUP2) was confirmed by histological study (Figure 1C,D). Nephroprotective treatment was started with ACE inhibitor (ramipril 2.5 mg/day). No relapse had occurred at the last follow‐up visit. His blood test 2 years after nephrectomy were as follow: creatinine clearance 148 mL/min/1.73 m^2^ (Cockroft‐Gault), no urinary albumin excretion, uricemia 0.290 mmoL/L, triglyceridemia 1.55 mmoL/L, lactatemia and glycemia normal before and after meals. Due to the rarity of this tumor and the young age of the patient, a genetic cause has been evoked but not confirmed to date (looking for a germline mutation of the fumarate hydratase gene).

## 3 PATIENT 2

**Figure 2:**
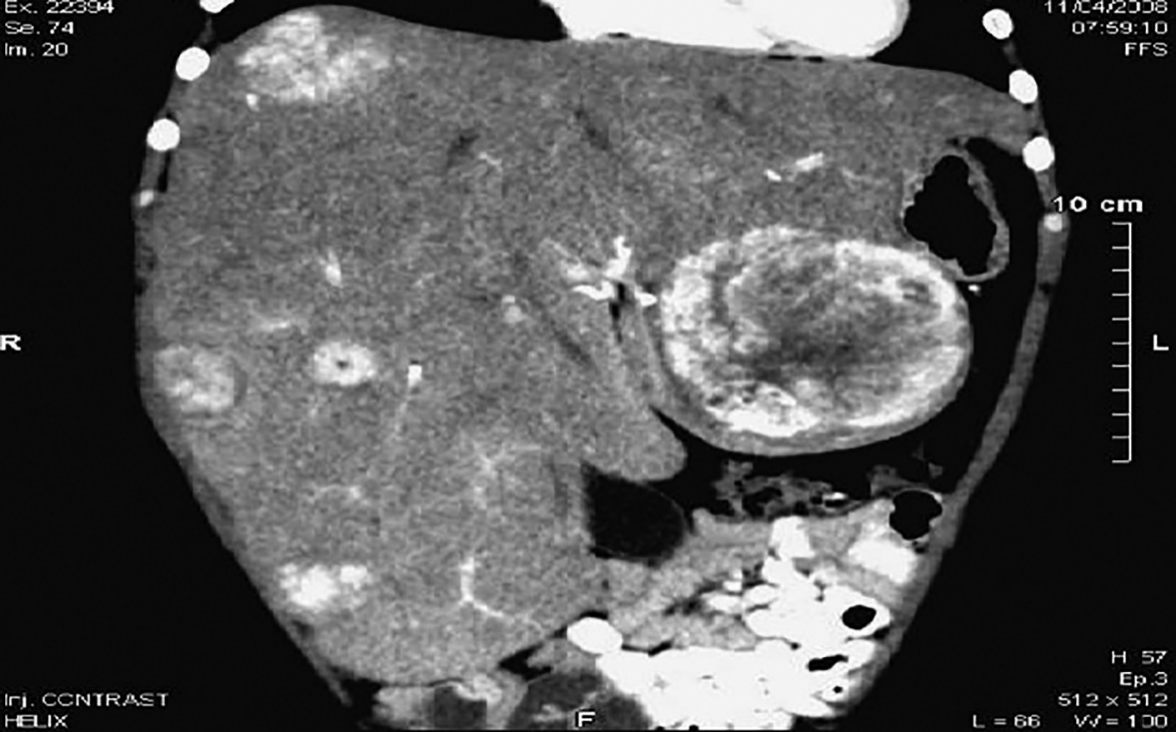
Liver MRI, multiple hepatic lesions, the largest of which is located in the left lobe of the liver and measures 10 cm, hyper T2 with heterogeneous enhancement after injection

We report on a 27‐year‐old female patient with a past history of GSD Ia, admitted for the diagnosis work up of a renal mass. Her parents were consanguineous, and she had two brothers (one died in the neonatal period from an unknown cause and one had GSD Ia diagnosed at the age of 5 months) and one healthy sister. She was diagnosed with GDS Ia at 3 weeks of life because of the family history. The identified homozygous mutation was c.79delC (p.Gln27fx). From the onset of the disease, she suffered from hepatomegaly and hypoglycemia, lactic acidosis, hypertriglyceridemia, and hyperuricemia after short fasts. Other metabolic complications included a prolonged bleeding time due to impaired platelet function, as well as an anemia due to iron deficiency, refractory to oral supplementation. She developed growth retardation with a final height of 151 cm. She was treated by a continuous enteral feeding until the age of 6 years and then, due to a poor metabolic control, nocturnal enteral feeding was resumed from the age of 13 to the age of 21. Since that age, carbohydrates account for 65% of caloric intake (350 g including 220 g of fractional ingestion of uncooked cornstarch). Under this diet, the metabolic parameters were as follow before breakfast: glycemia between 3.85 and 5.1 mmoL/L, lactic acid between 1.2 and 4.5 mmoL/L, bicarbonates between 15 and 23 mmoL/L, triglyceridemia between 3.42 and 6.84 mmoL/L, uricemia between 0.430 and 0.770 mmoL/L. The following long term complications were observed: hepatocellular adenoma increasing in number and size since the age of 12 (leading to surgical resection of a large suspect, painful and bleeding adenoma of 10 cm at the age of 23 which proved to be benign and without IL6ST, GNAS, STAT3, CTNNB1, HNF1A mutations (Figure 2), progressive renal dysfunction with a significant proteinuria between 0.7 and 0.9 g/24 h observed since the age of 20 without urolithiasis or nephrocalcinosis, despite hypocitraturia (0.5 mmol/day), frequent gout attacks, and osteoporosis. There were neither pancreatitis nor heart involvement.

**Figure 3:**
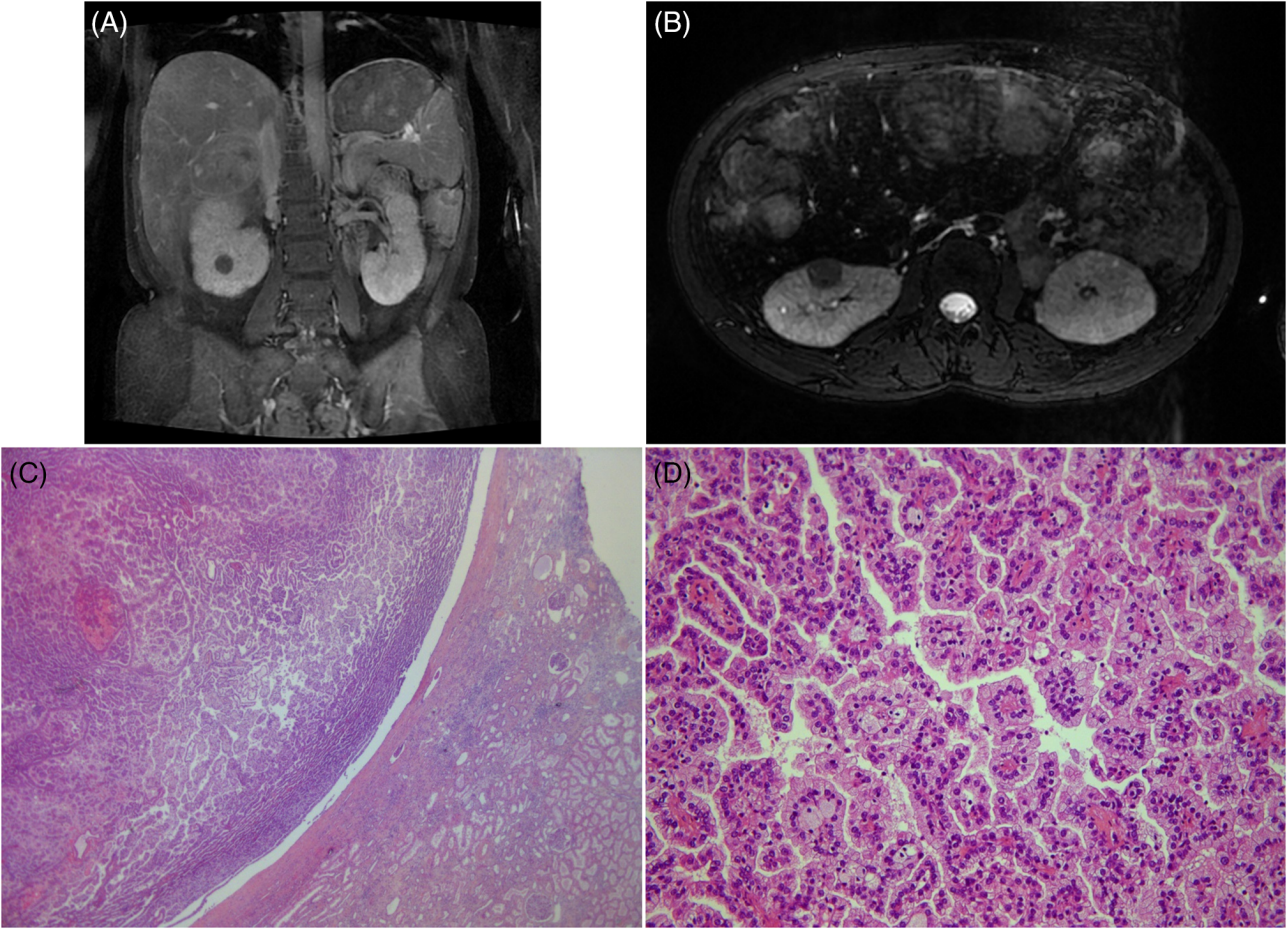
A, Renal MRI, right renal hypo T2 mass (diameter 16 mm). B, Renal MRI, right renal anterior hilar lip hypo T2 and poorly vascularized tumor (diameter 16 mm). C, Papillary renal tumor, HES ×25. D, Papillary renal cell carcinoma type 1, HES ×200

At the age of 27, a systematic yearly MRI to assess the progression of liver adenomas revealed the presence of a right renal anterior hilar lip hyper T1 and hypo T2 mass measuring 16 mm (Figure 3A,B). The retrospective analysis of the previous MRI, performed 1 year before, showed that this mass was already present and measured 9 mm at this time. A renal biopsy was performed after a 4‐day preparation to ensure an optimal metabolic control (intravenous glucose (4 mg/kg/min) with strict monitoring of blood glucose and lactate levels and then desmopressin injection before the procedure. Pathologic examination revealed a type 1, WHO/ISUP grade 2, papillary renal cell carcinoma. At this stage, serum creatinine was 96.8 μmol/L, EDTA/Cr51 clearance of the creatinine was 60 mL/min/1.73 m^2^ and proteinuria was 0.92 g/24 h despite treatment by ACE inhibitor.

A partial right nephrectomy was performed shortly after, with the same metabolic preparation. Pathologic examination revealed a 2 cm type 1, WHO/ISUP grade 2, renal cell papillary carcinoma (pT1aR0) (Figure 3C,D). The healthy kidney parenchyma analysis showed glomerular hypertrophy, with a few obsolescent glomeruli and lesions of focal segmental glomerulosclerosis. She experienced a right hemothorax due to a pleural breach, and a rise of serum creatinine to 167.2 μmol/L after surgery. Nine months after the surgery the EDTA/Cr51 clearance of the creatinine was 24 mL/min/1,73m^2^. Two years after partial nephrectomy, renal function had further worsened and chronic hemodialysis was started. Thirty‐two months later, the absence of relapse of the kidney cancer and its good prognosis, the good metabolic control and improved nutritional state under nocturnal enteral feeding allowed a combined liver‐kidney transplantation at the age of 32.

## 4 DISCUSSION

Kidney disease is a frequent complication of GSDI. Excessive accumulation of fat and glycogen in kidney leads to renal complications, such as hyperfiltration (70% of young adults) and proteinuria (40% of young adults), nephrocalcinosis due to hypercalciuria and hypocitraturia.^4^ Long‐term complications include interstitial fibrosis and focal segmental glomerulosclerosis. The appearance of renal cysts during the course of the disease has recently been described in the mouse model and in humans.^2^ In the GSDIa mouse model (KG6PC−/−), one mouse developed a clear cell renal carcinoma but it developed cyst prior to carcinoma. The pathophysiological mechanisms that may exist between elevation of G6P and a potential evolution to renal cysts are being investigated. These data suggest the possible occurrence of renal carcinoma in GSDI at an advanced stage of chronic kidney disease. Moreover, the downregulation of the tumor suppressor HNF1B observed in KG6pc−/− mice is known to predispose to renal cancer development.^6^

A single case of renal cancer in a GSDI patient has been reported so far in the literature. A 19‐year‐old female patient, diagnosed with GSDIb and chronic neutropenia, has developed a clear cell renal carcinoma. The patient had no renal cyst before. The authors discussed the role of GCSF for triggering this cancer, whereas no kidney cancer has been described in patients on GCSF therapy.^7^

For the first patient, carcinoma developed on “healthy” kidneys. The patient had no specific risk factor for kidney cancer, but active smoking. The second patient had severe kidney involvement, but without evident cyst on regular MRI and ultrasound follow‐up. In these two patients, parameters of metabolic control do not significantly differ from the other GSDIa patients of our center cohort.^2^

Finally, it is noteworthy that both patients were very young for developing kidney cancer, whereas the median age at diagnosis for this condition is 60 years and over.

## 5 CONCLUSION

To the best of our knowledge, these two reports are the first of kidney cancer in GSDIa patients. The origin of renal cancer is not yet understood. We suggest that annual monitoring of kidney imaging should be performed in GSD I patients from the second decade of life.

### PATIENT CONSENT STATEMENT

The authors have indicated that informed consent of patients was obtained. All procedures followed were in accordance with the ethical standards of the responsible committee on human experimentation (institutional and national) and with the Helsinki declaration of 1975, as revised in 2000.

## CONFLICT OF INTEREST

The authors declare that they have no conflict of interest.

## AUTHOR CONTRIBUTIONS

Dr Perry drafted the initial manuscript, and reviewed and revised the manuscript. Dr Douillard drafted the initial manuscript, and reviewed and revised the manuscript. Dr Hubert, Dr Jonca, Pr Glowacki and Pr Labrune critically reviewed the manuscript for important intellectual content, and revised the manuscript. Pr Leroy provided histologic figures for patient 2, and critically reviewed the manuscript. Dr Caveriviere provided histologic figures for patient 1, and critically reviewed the manuscript. All authors approved the final manuscript as submitted.
